# Mortality among long-stay patients with schizophrenia during the setting-up of community facilities under the Yuli model

**DOI:** 10.1080/21642850.2014.908717

**Published:** 2014-04-30

**Authors:** Kan-Yuan Cheng, Chih-Yuan Lin, Tzu-Kuei Chang, Chaucer C.H. Lin, Tsung-Hsueh Lu, Shu-Yuan Chen

**Affiliations:** ^a^ Department of Psychiatry, Yuli Veterans Hospital, Yuli, Taiwan, Republic of China; ^b^ Department of Public Health, Tzu Chi University, Hualien, Taiwan, Republic of China; ^c^ Medical Division, Eli Lilly and Company, Taipei, Taiwan, Republic of China; ^d^ Department of Psychiatry and Institute of Human Development, Tzu Chi University, Hualien, Taiwan, Republic of China; ^e^ Department of Public Health, College of Medicine, National Chen Kung University, Tainan, Taiwan, Republic of China

**Keywords:** schizophrenia, mortality, community care, Yuli model, reform

## Abstract

*Objective:* Over the past 15 years, Yuli Veterans Hospital (YVH) in Taiwan has developed the Yuli model to reform long-stay care for psychiatric patients. The development of the Yuli model could be divided into pre-early (1998–1999), early (2000–2006) and late (2007–2008) periods according to the setting-up of the community facilities. In the pre-early period, a vocational rehabilitation program was established for psychiatric patients in YVH. In the later periods, the independent living skills training and the program for social reintegration were instituted in the community facilities. This study aimed to evaluate mortality among the long-stay patients with schizophrenia during the three periods. *Methods:* In all, 2457 patients with schizophrenia who had been hospitalized for at least one year initially were retrospectively followed from 1 January 1998 to 31 December 2008. Compared with the general population in Taiwan, we calculated the age- and sex-specific standardized mortality ratios (SMRs) of those patients by cause of death during the three periods. *Results:* Most of the patients were male (81.3%). The mean ± SD age of the patients was 57.83 ± 16.95 years. The all-, natural- and unnatural-cause mortalities of the patients were nearly two times greater than those of the general population during the whole study period. Compared with those in the pre-early and early periods, all patients in the late period had the lowest mortality gaps. In the pre-early, early and late periods, the all-cause SMR were 5.40 (95% confidence interval (CI) = 4.27–6.81), 2.90 (95% CI = 2.20–3.79) and 1.17 (95% CI = 0.54–2.22), respectively, for the 50–69-year-old male patients. Nearly half of all the patients who participated the whole comprehensive rehabilitation program belonged to this sex and age group (*N* = 156, 46.6%). *Conclusions:* With the setting-up of community facilities for the comprehensive rehabilitation program, the mortality gaps among the 50–69-year-old male patients apparently decreased using the Yuli model.

## Introduction

Long-stay patients with schizophrenia have higher natural and unnatural death rates than the general population (Harris & Barraclough, 1998; Licht, Mortensen, Gouliaev, & Lund, 1993; Rasanen, Hakko, Viilo, Meyer-Rochow, & Moring, 2003). In countries with deinstitutionalization, long-stay patients with mental illness are no longer in psychiatric hospitals and have received care from community rehabilitation services since the 1960s or 1970s. Several studies have revealed improvements in the death rates among long-stay patients with mental illness (Craig & Lin, 1981; Pirkola, Sohlman, Heila, & Wahlbeck, 2007; Rantanen et al., 2009), but other studies have reported that mortality among long-stay patients with mental illness was not associated with the process of deinstitutionalization or that mortality gaps still existed between those patients and the general population in the post-deinstitutionalization period (D'Avanzo, La, Vecchia, & Negri, 2003; Grigoletti et al., 2009; Salokangas, Honkonen, Stengard, & Koivisto, 2002). The reported changes in mortality seem to be inconsistent among long-stay patients with mental illness with the shifting of care from the inpatient setting to community facilities.

Yuli Veterans Hospital (YVH) in Yuli town, Hualien County in eastern Taiwan, is a psychiatric teaching hospital that accommodates patients with severe mental disability from all over Taiwan. Most of the patients who receive long-stay care at YVH have suffered from schizophrenia for many years and have experienced the collapse of family or social support in the original community (Lin, Huang, Minas, & Cohen, 2009). The psychiatric facilities in YVH include two acute wards, four chronic wards, five nursing home wards, one-day ward, one recovery home and three community rehabilitation centers with a total capacity of 3619 beds. As a general hospital with a 101-bed capacity, YVH also provides general medical care for the local residents in southern Hualien and long-stay patients with mental illness, except for those with acute cardiovascular events, severe traumas or cancers, who need to be transferred to the medical center in Hualien City. During the past 15 years, the health delivery in general care has not changed significantly at YVH. However, YVH has reformed psychiatric care for long-stay patients with mental illness and established the Yuli model with the support of the national budget and payments by the National Health Insurance program in Taiwan for services provided in community facilities (Akiyama et al., 2008; Lin et al., 2009). In 1998, the vocational rehabilitation program, including occupational therapy, industrial therapy, prevocational training and sheltered or supported employment, was established in YVH. In 2000, a recovery home with a 280-bed capacity was set up to offer patients with mental disability independent living skills training in well-arranged physical environments and supported employment in Yuli town (Lin et al., 2009). In line with the concept of Yuli town being a therapeutic community, YVH set up two community rehabilitation centers with a total capacity of 220 patients in Yuli town in November of 2006. The psychiatric rehabilitation team promotes social reintegration for patients with mental disability recovering with the collaboration of local civil organizations (Lin et al., 2009). Based on the timing of the establishment of these community facilities, we can divide the reform process into pre-early (1998–1999), early (2000–2006) and late (2007–2008) periods.

Mortality should be viewed as one of the major outcomes of patients with schizophrenia under any health care plan (Isaac, Chand, & Murthy, 2007). However, few studies have focused on mortality among patients with schizophrenia during the reformation of long-stay care in countries without deinstitutionalization. The aim of this study was to evaluate the changes in the mortality gap between long-stay patients with schizophrenia and the general population in Taiwan during the three periods in the reform process of psychiatric care in YVH.

## Methods

### Study sample

In a search of the inpatient registration digital database of YVH, we found 3122 patients aged at least 20 years that had been admitted to the chronic or nursing home psychiatric wards at YVH between 1995 and 1997. The first admission diagnoses of these 3122 patients included schizophrenia (*International Classification of Diseases, 9th Revision* (*ICD-9*) 295, *n *= 2887, 92.5%), organic mental disorder (*ICD-9* 294, *n *= 112, 3.6%), dementia (*ICD-9* 290, *n *= 71, 2.3%), affective disorder (*ICD-9* 296, *n *= 32, 1.0%) and others (*n *= 20, 0.6%). In the patients with a first admission diagnosis of schizophrenia, we excluded those who had been hospitalized for less than one year and died in or left YVH before 31 December 1997. All 2457 eligible patients were retrospectively followed from 1 January 1998 to 31 December 2008. To confirm the direct cause of death and the death date, we linked the National Death Certificate System in Taiwan with the identity numbers of the eligible patients. A death event was defined as: (i) the death of an eligible patient in any facility of YVH and (ii) the death of an eligible patient after transferring to another hospital for advanced medical treatment. The end point of follow-up in the patients who died was the death date as recorded in the National Death Certificate System in Taiwan. Since the aims of this study were to evaluate the mortality of those patients who had experienced the entire reform of the psychiatric care program in YVH, discharged patients and others who experienced an interruption in hospitalization for more than 30 days were identified as withdrawals. Patients who were transferred or referred to other facilities were also considered as withdrawals, except for those with death events. The end points of follow-up for the withdrawals and survivors were the discharge dates and 31 December 2008, respectively.

The characteristics of the subjects, including demographic data and psychiatric history, were recorded. All of the data were retrieved from the inpatient registration digital database of YVH. The welfare status was based on the resource of social security in Taiwan. The Veterans Affairs Commission provides the veterans the all welfares and the veterans' relatives with mental handicaps some assistance. The district governments of census registration give low-income patients a monthly subsidy. Patients who have a hospital address as their census registration may have no family, or their family does not want the patient to use the same address in the census registration. In terms of psychiatric history, a complete rehabilitation program includes the following services: holistic psychiatric and medical support, vocational rehabilitation, case management and a residential program under the Yuli model (Lin et al., 2009). The patients who lived in the recovery home of YVH should have received the above services, and therefore living in the recovery home for at least six months could be taken as indicating that the patient had participated in the whole comprehensive rehabilitation program.

### Classifications of causes of death and calculation of mortality

The National Death Certificate System in Taiwan used the *ICD-9* to categorize the causes of death in 1998–2007 and the *Classification of Diseases, 10th Revision* (*ICD-10*) in 2008, so we converted the ICD-10 codes to ICD-9 codes for the causes of death of patients who died in 2008. All causes of death were initially categorized as natural (*ICD-9* 001–799) and unnatural (E800–E999). Then, the classifications of natural causes of death were based on the major ICD-9 codes according to the physiopathology, affected organs or physiological systems. Unnatural causes of death included three major subgroups, accidents (E800–E949), suicide (E950–E959) and undetermined external causes (E980–E989).

The standardized mortality ratios (SMRs) were the ratio of the number of observed deaths in this study cohort to the number expected in the general population of Taiwan. The numbers of observed deaths and corresponding cumulative person-years in this study cohort were collected according to the actual ages of the study subjects in each year of follow-up. The numbers of expected deaths each year were initially calculated by the crude death rates of the general population in Taiwan multiplied by the corresponding number of cumulative person-years in every 10-year age interval. All of the above numbers of expected deaths were merged into the same age-and-sex group to be divided by the corresponding number of observed deaths in the three periods of this study and the causes of death. The 95% confidence interval (95% CI) of the SMR was estimated by following a Poisson distribution (Haenszel, Loveland, & Sirken, 1962). All of the above study procedures were approved by the Institution Review Board of YVH.

## Results

### Characteristics of the subjects

The age (mean ± SD) of the study subjects was 57.83 ± 16.95 years. A total of 1998 patients were male (81.3%) and 2124 patients were unmarried (86.4%). Two out of three patients were veterans (*n *= 1652, 67%). Nearly half of all patients had YVH as their address in their census registration (*n *= 1183, 48%). Before follow-up, most of the eligible patients had lived in YVH for at least two years (the 25th percentile of the hospitalization period initially was 1.98 years). The median duration of follow-up was 10 years. Only 102 patients (4%) had a combined psychiatric diagnosis other than schizophrenia. In all, 335 patients (13.6%) had participated in the whole comprehensive rehabilitation program. Of this group, 156 (46.6%) were male and aged 50–69 years, 90 (26.9%) were female and aged at least 20 years, 76 (22.7%) were male and aged 20–49 years and only 13 (3.9%) were male and aged at least 70 years. The follow-up results showed that 1184 (48.2%) patients had persistently received care in YVH, 993 (40.4%) had died in YVH or after transferring for advanced medical treatment and 280 (11.4%) had withdrawn (Table 1).

**Table 1.** Characteristics of 2457 long-stay patients with schizophrenia at YVH and the results of follow-up from 1998 to 2008.

Characteristics/results of follow-up	*N*	%
Demography		
Age (years), mean ± SD	57.83 ± 16.95	
Sex		
Male	1998	81.3
Female	459	18.7
Marital status		
Unmarried	2124	86.4
Married	197	8
Widowed	98	4
Divorced	38	1.5
Welfare status^a^		
Veterans	1652	67.2
Relatives of veterans	561	22.8
Low income	131	5.3
General	113	4.6
Census registration		
Hospital^b^	1183	48.1
Non-hospital	1274	51.9
Psychiatric history		
Hospitalization duration in initial follow-up (years), median (25–75th percentiles)	2.84 (1.98–10.78)	
Follow-up duration (years), median (25–75th percentiles)	9.98 (3.94–11.00)	
Combined with other psychiatric diagnoses	102	4.2
Participation in the whole comprehensive rehabilitation program^c^	335	13.6
Results of follow-up		
Under the care of YVH	1184	48.2
Inpatient settings	1008	41
Community facilities	176	7.2
Death	993	40.4
In YVH	670	27.3
After transferal	323	13.1
Withdrawals	280	11.4
Long-stay in other facilities	156	6.3
Return home	47	1.9
Other^d^	77	3.1

^a^The classifications based on social welfare resources in Taiwan.

^b^Those with no family or their family did not want the patient to use the same address in registration census.

^c^The services of holistic psychiatric and medical support, vocational rehabilitation, case management and a residential program under the Yuli model.

^d^Referral to outpatient department (57 persons), discharge against advice (14 persons) and leaving without leaving a message (6 persons).

### Causes of death and cause-specific SMRs

Most of the decedents died of natural causes (*N* = 942, 94.9%). Diseases of the respiratory system (*ICD-9* 460–519) (*N* = 232, 23.4%) were the leading cause of death, followed by diseases of the circulatory (390–459) (*N* = 151, 15.2%) and digestive systems (520–579) (*N* = 108, 10.9%). Forty-three patients (4.3%) died due to accidents. Only four patients (0.4%) died by suicide. A total of 18,649.8 person-years were followed, and the all-cause SMR was 2.17, with a 95% CI of 2.04–2.21. The natural- and unnatural-cause death rates of the eligible patients were each approximately two times greater than those in the general population. For most natural causes except neoplasms (140–239), all patients had a significantly higher risk of death than the general population (SMR = 0.66, 95% CI = 0.53–0.83). The patients also had a significantly higher death rate from accidents (SMR = 2.28, 95% CI = 1.65–3.07); however, the suicide death rate among the patients was similar to that in the general population (SMR = 0.70, 95% CI = 0.19–1.80) (Table 2).

**Table 2.** Distribution of causes of death among 993 decedents and SMRs by causes of death among 2457 long-stay patients with schizophrenia at YVH from 1998 to 2008.

Cause of death (ICD-9)	*N*	(%)	Expected deaths	SMR^a^	95% CI
All causes	993	(100.0)	456.9	2.17	2.04–2.31
Natural causes (001–799)	942	(94.9)	431.2	2.18	2.05–2.33
Diseases of respiratory system (460–519)	232	(23.4)	63.0	3.68	3.23–4.20
Diseases of circulatory system (390–459)	151	(15.2)	107.2	1.41	1.20–1.64
Diseases of digestive system (520–579)	108	(10.9)	32.3	3.34	2.75–4.05
Diseases of genitourinary system (580–629)	89	(9.0)	21.2	4.20	3.39–5.20
Symptoms, signs, and ill-defined conditions (780–799)	83	(8.4)	31.0	2.68	2.15–3.34
Neoplasms (140–239)	80	(8.1)	120.6	0.66	0.53–0.83
Infectious and parasitic diseases (001–139)	66	(6.7)	13.9	4.75	3.66–6.10
Mental disorders (290–319)	51	(5.1)	1.9	26.84	20.00–35.39
Endocrine, nutritional and metabolic diseases, and immunity disorders (240–279)	29	(2.9)	29.8	0.97	0.65–1.40
Diseases of the skin, and subcutaneous tissues (680–709)	29	(2.9)	2.4	12.08	8.10–17.40
Diseases of musculoskeletal system and connective tissue (710–739)	15	(1.5)	1.7	8.82	4.94–14.55
Diseases of nervous and sense organs (320–389)	6	(0.6)	5.1	1.18	0.43–2.56
Diseases of blood and blood-forming organs (280–289)	3	(0.3)	1.1	2.73	0.56–7.97
Unnatural causes (E800–E959)	51	(5.1)	25.6	1.99	1.48–2.62
Accidents (E800–E949)	43	(4.3)	18.9	2.28	1.65–3.07
Suicide (E950–E959)	4	(0.4)	5.7	0.70	0.19–1.80
Undetermined external causes (E980–E989)	4	(0.4)	1.1	3.64	0.99–9.32

^a^Compared with the general population of Taiwan from 1998 to 2008.

### SMRs by age and sex groups and the three periods with the reform

The all-cause SMRs decreased slightly by period, but significant mortality gaps still existed in all periods. Within all age and sex groups, the 50–69-year-old males had the highest all-cause SMR, 5.40 (95% CI = 4.27–6.81), in the pre-early period, but the lowest SMR, 1.17 (95% CI = 0.54–2.22), in the late period. Only male patients aged at least 70 years still had a significantly higher all-cause death rate than the general population in the late period (Table 3). The age- and sex-specific SMRs from natural causes during the three periods showed that the 50–69-year-old males and the females aged at least 20 years both had decreased mortality gaps over the three periods (Table 4). The patients in the four age and sex groups did not have significantly higher unnatural death rates than the general population in the late period. The SMRs of unnatural causes among all patients in the early and late periods (1.76 and 1.14, respectively) were lower than those in the pre-early period (2.96). There were no unnatural deaths during the late period among the 50–69-year-old male patients (Table 5).

**Table 3.** SMRs of all causes of deaths among 2457 long-stay patients with schizophrenia at YVH by age and sex groups and the three periods of the reform of psychiatric care.

Age and sex groups (years)	%	Pre-early (1998–1999)				Early (2000–2006)				Late (2007–2008)			
		O	E	SMR^a^	(95% CI)	O	E	SMR^a^	(95% CI)	O	E	SMR^a^	(95% CI)
All	100.0	306	109.4	2.80	(2.50–3.14)	529	264.3	2.00	(1.82–2.18)	158	83.2	1.90	(1.62–2.23)
Female (≥20)	18.7	17	6.0	2.83	(1.65–4.53)	31	14.4	2.15	(1.46–3.06)	11	6.3	1.75	(0.87–3.13)
Male (20–49)	23.5	9	2.8	3.21	(1.47–6.10)	18	6.3	2.85	(1.69–4.45)	1	0.7	1.43	(0.04–7.97)
Male (50–69)	25.1	74	13.7	5.4	(4.27–6.81)	56	19.3	2.9	(2.20–3.79)	9	7.7	1.17	(0.54–2.22)
Male (≥70)	32.7	206	86.8	2.34	(2.04–2.69)	424	224.3	1.89	(1.72–2.08)	131	67.0	1.96	(1.64–2.33)

Notes: O, observed number of decedents and E, expected number of decedents.

^a^Compared with the general population of Taiwan.

**Table 4.** SMRs of deaths by natural causes among 2457 long-stay patients with schizophrenia at YVH by age and sex groups and the three periods of the reform of psychiatric care.

Age and sex groups (years)	%	Pre-early (1998–1999)				Early (2000–2006)				Late (2007–2008)			
		O	E	SMR^a^	(95% CI)	O	E	SMR^a^	(95% CI)	O	E	SMR^a^	(95% CI)
All	100.0	285	102.3	2.79	(2.48–3.14)	504	250.1	2.02	(1.85–2.21)	153	78.8	1.94	(1.65–2.28)
Female (≥20)	18.7	16	5.5	2.91	(1.66–4.71)	25	13.4	1.87	(1.21–2.77)	9	5.9	1.53	(0.70–2.91)
Male (20–49)	23.5	8	1.8	4.44	(1.91–8.75)	15	4.2	3.57	(2.00–5.89)	6	1.5	4.00	(1.47–8.72)
Male (50–69)	25.1	71	12.2	5.82	(4.57–7.38)	53	17.3	3.06	(2.30–4.02)	9	7	1.29	(0.59–2.45)
Male (≥70)	32.7	190	82.8	2.29	(1.98–2.65)	411	215.1	1.91	(1.73–2.11)	129	64.5	2.00	(1.68–2.39)

^a^Compared with the general population of Taiwan.

**Table 5.** SMRs of deaths by unnatural causes among 2457 long-stay patients with schizophrenia at YVH by age and sex groups and the three periods of the reform of psychiatric care.

Age and sex groups (years)	%	Pre-early (1998–1999)				Early (2000–2006)				Late (2007–2008)			
		O	E	SMR^a^	(95% CI)	O	E	SMR^a^	(95% CI)	O	E	SMR^a^	(95% CI)
All	100.0	21	7.1	2.96	(1.83–4.53)	25	14.2	1.76	(1.14–2.60)	5	4.4	1.14	(0.37–2.66)
Female (≥20)	18.7	1	0.5	2	(0.05–11.14)	6	1	6	(2.20–13.08)	2	0.4	5	(0.61–18.05)
Male (20–49)	23.5	1	1.1	0.91	(0.02–5.07)	3	2	1.5	(0.31–4.38)	1	0.7	1.43	(0.04–7.97)
Male (50–69)	25.1	3	1.5	2	(0.41–5.84)	3	2	1.5	(0.31–4.38)	0	0.8	0	–
Male (≥70)	32.7	16	4	4.0	(2.29–6.48)	13	9.2	1.41	(0.75–2.41)	2	2.5	0.8	(0.10–2.89)

^a^Compared with the general population of Taiwan.

## Discussion

### Excess mortality and leading causes of death

The long-stay patients with schizophrenia still had higher death rates than the general population during the whole study period. Before the introduction of the National Health Insurance program in Taiwan, Chen and colleagues evaluated the five-year mortality of 7163 male and 2876 female schizophrenia inpatients hospitalized from from 1987 to 1988 (Chen, Huang, Yeh, Rin, & Hwu, 1996). Compared with the general population, the all-cause SMRs of the male and female patients were 2.7 and 4.7 (Chen et al., 1996), respectively, and the mortality gaps were greater than those in our study (2.17 and 2.21, respectively). The differences in the mortality gap might be related to the provision of both physical and mental health care for long-stay schizophrenia patients through Taiwan's National Health Insurance. In an eight-year follow-up study of long-stay patients with major schizophrenia in northern Finland, the all-cause SMRs were 4.0 and 4.1 in the 157 male and 96 female patients, respectively (Rasanen et al., 2003). Nevertheless, the mortality gaps for accidents and suicide (SMR = 5.1 and 5.9) in the Finland study (Rasanen et al., 2003) were much larger than those in our results (2.3 and 0.7, respectively). Only 16.6% of all patients in our study lived in community facilities or returned home, but 63.2% of the patients in the Finland study left the psychiatric hospital (Rasanen et al., 2003). The different mortality gaps in the Finland study and our study might be explained by the varied risks of unnatural death in different living placements.

Diseases of the respiratory, circulatory and digestive system were the leading causes of death in our study (Table 2). The vulnerability to respiratory diseases in patients with schizophrenia (Copeland et al., 2007; Filik et al., 2006) could be related to the higher proportion of patients who smoke (McCreadie, 2003) and to exposure to antipsychotics (Barnett, Perry, Alexander, & Kaboli, 2006). Smoking, an unhealthy diet and the adverse effects of antipsychotics have also been frequently reported to be associated with cardiovascular diseases in patients with mental illness (Henderson et al., 2005; Joukamaa et al., 2006; McCreadie, 2003). The explanation for the excess mortality from digestive diseases (Brown, 1997; Licht et al., 1993; Rasanen et al., 2003) could be the under-detection (Brown, Inskip, & Barraclough, 2000) of gastrointestinal symptoms or signs, the risk of viral hepatitis infections (Goff et al., 2005) and the harmful use of alcohol. To prevent the complication of invasive pneumonia, regular influenza and pneumococcus vaccines are recommended for patients with schizophrenia (Goff et al., 2005). In addition, evaluation of the common vulnerability to the three major systematic diseases could be important for inpatients with schizophrenia due to the consistent excess mortality from these diseases (D'Avanzo et al., 2003; Harris & Barraclough, 1998; Rasanen et al., 2003). The result of excess mortality from mental illness among all patients was supported by the findings of a previous Italian study (D'Avanzo et al., 2003). Furthermore, the underlying dysfunctions of the autonomic nervous system or the potential lethal effects of antipsychotics might be considered as the death mechanism in patients who died of schizophrenia (Rosh, Sampson, & Hirsch, 2003).

All eligible patients had a significantly lower death rate from neoplasms than the general population (SMR = 0.66, 95% CI = 0.53–0.83). Under-detection (Brown et al., 2000; Felker, Yazel, & Short, 1996) and bias due to competing causes of death (Guan et al., 2013) might be explanations for the relatively low death rates from cancer in these patients. However, owing to the practice of regular pap smears and mammographic examinations for female inpatients with mental illness in YVH, only one female patient died of cervical cancer in our study.

### The changes in mortality gaps following the reform of psychiatric care

The mortality gaps from all, natural and unnatural causes between the 50–69-year-old male patients and the general population apparently decreased over the three periods (Tables 3 4–5). This group had the highest proportion of patients (*N* = 156, 46.6%) to experience the whole comprehensive rehabilitation program of the four age and sex groups. However, the male patients aged at least 70 years and the 20–49-year-old male patients both still had significantly higher natural-cause death rates in the late period (Table 4). Only 13 (3.9%) and 76 patients (22.7%) in these two age and sex groups experienced the whole comprehensive rehabilitation program. Therefore, the proportions of those experiencing the whole comprehensive rehabilitation program might be associated with the changes in the all- or natural-cause mortality gaps in the different age and sex groups during the period of the establishment of community facilities with the reform of psychiatric care.

The mortality gap for death from unnatural causes between all eligible patients and the general population decreased apparently over the three periods of reform (Table 5). Our results are supported by the findings of a previous study of specific populations that received mental health care during the reform of psychiatric services (Bulow, Svensson, & Hansson, 2002; Farragher, Carey, & Owens, 1996; Pirkola et al., 2007). However, some previous studies showed a persistent excessive mortality due to unnatural causes during the periods of deinstitutionalization (D'Avanzo et al., 2003; Rasanen et al., 2003). In a cohort of patients with mental illness, the lower unnatural-cause death rate in the later evaluated period could be explained by the fact that the patients with mental illness were at greater risk of death by suicide during the earlier hospitalization period or at initial discharge (Chen et al., 1996; Hansen, Jacobsen, & Arnesen, 2001). However, our study subjects were all hospitalized for at least one year in the initial follow-up and those patients were also encouraged to leave the inpatient setting to receive care in community facilities. With rehabilitation programs in the community, psychiatric patients might have more motivation to maintain both mental and physical well-being owing to competition in employment or striving for a better financial status or social relationships. We supposed that the process of social reintegration with the rehabilitation program might provide protection from the risks of suicide and accidents.

We should consider the effects of the changes in the death rate in the general population in Taiwan from 1998 to 2008. The decreased gaps in mortality by unnatural causes from the pre-early to the early period could be partially due to the elevated suicide rates in the general population in Taiwan from 1998 to 2006 (Ministry of Health and Welfare in Taiwan, 2014). The gaps in unnatural-cause mortality continued to decrease in the late period when the suicide rates in the referent population had dropped in 2007. However, the natural-cause death rates in the general population in Taiwan persistently decreased from 1998 to 2008 (Ministry of Health and Welfare in Taiwan, 2014). The decreased gaps in mortality by natural causes in the early or late period should be mainly due to the effects of the reform of psychiatric care. Selection bias could be considered here, because those that withdrew were significantly younger (55.6 ± 18.0 vs. 58.1 ± 16.8, *t *= 2.2, *P *= 0.03) and a lower proportion of those registered in the hospital than the patients who had continually received the care by YVH (*n *= 115, 41.1% vs. *n *= 1068, 49.1%, *χ* ^2 ^= 6.3, *P *= 0.01). But the withdrawals had a higher death rate than the patients who had persistently received care at YVH from 1998 to 2008 (*n *= 150, 53.6% vs. *n *= 993, 45.6%, *χ* ^2 ^= 6.0, *P *= 0.01). Compared with the withdrawals that left in the pre-early period, the patients that left YVH in the early and late periods included a significantly higher proportion of those returning home or being referred to the outpatient department (*n *= 77, 47.3% vs. *n *= 27, 23.1%, *χ* ^2 ^= 16.0, *P *< 0.01) and had a lower death rate (*n *= 74, 45.4% vs. *n *= 79, 67.5%, *χ* ^2 ^= 12.6, *P *< 0.01). The withdrawals who returned home or were referred to the outpatient department had a clearly lower death rate than those who were relocated to other long-stay facilities (*n *= 16, 15.4% vs. *n *= 121, 77.6%, *χ* ^2 ^= 94.3, *P *< 0.01). Therefore, the patients who left YVH in the later periods might have had a greater likelihood of returning home or being referred to an outpatient department with better outcomes in mortality. The above findings could be compatible with the results of a decrease in the mortality gaps between the study cohort and the general population in Taiwan in the later periods.

### Strengths and limitations of the study

This study may be the first to evaluate the changes in mortality among schizophrenia patients with the reform of long-stay care in countries without deinstitutionalization. There are several strengths to this study. The study sample represented the entire target population, with a relatively low proportion of withdrawals (11.4%). The validity of the data from the inpatient registration digital database of YVH was satisfactory, because only two patients had an unmatched birth date or death date in the linkage with the National Death Certificate System in Taiwan. However, lacking group data on lifestyles and use of psychiatric medications is a limitation in our study. Furthermore, the establishment of community facilities could directly represent the shifting of therapeutic placements, but does not include the entire reform of the mental health service.

### Conclusion

In this retrospective observational study, we examined the 11-year mortality of a cohort of patients with schizophrenia under the reform of long-stay care in YVH. The decreased mortality gaps in the age and sex groups with relatively high proportions experiencing the whole comprehensive rehabilitation program is one of the positive long-term consequences of the increased provision of community facilities. In terms of the decreased gaps in mortality due to unnatural causes, social reintegration in the local community of the long-stay facility at Yuli might offer protection against accidents and suicide. The major implication of our study results could be limited to the subgroup of long-stay patients with chronic schizophrenia who might receive psychiatric rehabilitation during their middle age. Although those patients could have long-term mental disability and a collapse of family support, our study results support the promotion of community programs for them under the coverage of social welfare programs and the introduction of national health insurance in countries without deinstitutionalization. Based on our findings, the relationship between protection from death and the participation in psychiatric rehabilitation among patients with schizophrenia still requires further evaluation.

## Disclosures of interest

Dr Chaucer Lin is the Medical Director, a full time employee, of Eli Lilly and Company, Taiwan. The remaining authors have no interests to disclose.
